# Comparison of oral health-related quality of life in a group of 19-year-old patients with or without previous orthodontic treatment

**DOI:** 10.2340/aos.v85.45522

**Published:** 2026-03-04

**Authors:** Maryam Sonono, Linda Yousef, Sinan Dehrab, Marie Pegelow

**Affiliations:** Division of Orthodontics, Department of Dental Medicine, Karolinska Institutet, Huddinge, Sweden

**Keywords:** adolescents, malocclusion, QoL, orthodontic treatment, patient-reported outcomes

## Abstract

**Objective:**

To evaluate the impact of orthodontic treatment on the oral health-related quality of life (OHRQoL) several years after orthodontic treatment.

**Material and methods:**

Thirty-two patients with previous orthodontic treatment and 31 patients without previous orthodontic treatment, aged 19 years, were included in the study. A questionnaire consisting of three parts was used: The Oral Health Impact Profile (OHIP-S14), Jaw Functional Limitation Scale (JFLS-20) and Orofacial Aesthetic Scale (OES). Previous orthodontic treatment, malocclusions and earlier dental treatment were registered.

**Results:**

There were statistical differences between the orthodontic group and the control group regarding the total OHIP-S14 (*p* = 0.03) and the total OES (*p* = 0.028), as subjects in the control group indicated less satisfaction. There were non-statistical differences between the two groups regarding the JFLS-20. When divided into the four domains of OHIP-14, only the psychosocial domain showed a statistically significant difference between the groups (*p* = 0.006). No association was found between the decayed, missing and filled teeth index and total OHIP, total JFSL or total OES in both groups.

**Conclusion:**

Nineteen-year-old individuals who had undergone orthodontic treatment have a significantly better OHRQoL when compared to individuals without need for orthodontic treatment, specifically in the psychosocial and aesthetic domains.

## Introduction

Malocclusion is one of the most common oral health disorders. It is not considered a disease, but it can lead to reduced function of chewing, speech and swallowing. The aetiology of malocclusion is most commonly a combination of genetic and environmental factors [1]. The prevalence of malocclusion among Swedish children is approximately 71% although some types of malocclusions may self-correct as children transition from primary to permanent dentition [2].

The quality of life (QoL) can be affected by poor oral health. Oral health-related quality of life (OHRQoL), which is commonly assessed using questionnaires for dental research, is recognised by the World Health Organization (WHO). Functional health, social health, emotional well-being and economical associations are considered in oral health programs. The well-being of an individual can, among other things, be influenced by self-consciousness regarding the shape and discolouration of the teeth [3].

The Oral Health Impact Profile (OHIP) is a contributory validated factor in the measurement of OHRQoL, and there are three different versions of OHIP (-5, -14 and -49), with the OHIP-49 being the original one [4]. OHIP-14, which is a shortened and validated version of OHIP, is composed of 14 questions [5]. It is divided into four domains: oral function (F), orofacial appearance (A), orofacial pain (P) and psychosocial impact (PI) [6]. In this study, the Swedish version OHIP-S14 was used, which has demonstrated good reliability and acceptable validity [7]. The Jaw Functional Limitation Scale (JFLS-20) consists of 20 questions related to functional limitations of the jaw, related to mastication, vertical jaw-mobility and social and emotional well-being [8]. The Orofacial Aesthetic Scale (OES) consists of seven questions measuring how subjects evaluate their orofacial aesthetics, with an additional question for the overall value. The validity and reliability of the OES have been previously researched [9].

The age of the patient affects the impact of the malocclusion on the OHRQoL. Children under 8 years of age have a low correlation between malocclusion and QoL, while an impact is more noticeable on adolescents between the age of 11 and 14 years. The largest, yet still small, impact of malocclusion is seen in adolescents older than 14 years old. This indicates that the effect of their malocclusion increases as they get older [10].

Self-esteem is a factor that affects QoL [11, 12]. Taibah and co-authors [12] observed that male adolescents have a higher self-esteem compared to female adolescents. Findings from the study were that students who have finished orthodontic treatment seem to have a slightly higher self-esteem compared to untreated students. Severe malocclusions that have a greater impact on lip closure, speech and mastication will have a negative outcome on self-esteem and social interaction. Spacing of the incisors, crowding and overjet appear to be the types of malocclusions that primarily lead to a lower self-esteem [12, 13]. A Swedish study investigating the association between self-perceived orthodontic treatment need in children of different geographic origins showed that 12–13-year-old adolescents who believe that they need orthodontic treatment also have a need according to the Index of orthodontic treatment need (IOTN)-Dental health component (DHC); an association was found between these subjects and IOTN-DHC grades 4 and 5 [14].

Treatment outcome can be evaluated on dental cast comparing before and after treatment with several indices, for example the Peer Assessment Rating index (PAR). It has been shown that bimaxillary fixed appliances treatment by orthodontic specialists produced the highest quality of orthodontic outcomes in a study from Southeast Wales [15].

The design of this study was to give questionnaires to a group of previously treated 19-year-old Swedish adolescents to investigate whether they still value the orthodontic treatment compared to a group that has not received treatment. The Swedish Dental Board index was used as tool for assessing the orthodontic treatment need [16–18]. It consists of a four-grade index scale that describes the priority of need for orthodontic treatment: very urgent need (grade 4), urgent need (grade 3), moderate need (grade 2) and little need (grade 1) [18]. The different malocclusions included in each grade are described in the English version of the Swedish Dental Board index [18] (Supplementary Appendix). The IOTN index was developed as a modification of the index of treatment by the Swedish Dental Board, but it features measurable distinctions between the levels [17]. The Swedish Dental Board index and the IOTN are orthodontic treatment need indices that also evaluate how the patient feels about their oral appearance. Since the Swedish Dental Board index also addresses the patient’s motivation for orthodontic treatment, it can also be associated to the patient’s well-being and QoL. The age of the participants was set to 19 because they finished their orthodontics treatment several years ago, which might lead to a better understanding and self-awareness of their oral health.

### Aims and hypothesis

The aim was to evaluate whether previous orthodontic treatment has a positive effect on the OHRQoL several years after treatment.

Our null hypothesis is that 19-year-old patients who have undergone orthodontic treatment earlier will have no difference in their OHRQoL than those who have not received any orthodontic treatment.

## Materials and methods

### Subjects

This study includes patients (*n* = 63) from Huddinge, Sweden. Thirty-one of the participants were in the control group, whereas 32 participants were in the orthodontic group. Ethical approval for the study was granted by the Regional Ethical Review Board in Stockholm (2017/874-31).

Inclusion criteria for the orthodontic group were adolescents in the permanent dentition, 19 years of age, with completion of their orthodontic treatment. The age of the participants was set at 19 years because they had completed their orthodontic treatment several years prior, which was expected to contribute to greater understanding and self-awareness of their oral health. Inclusion criteria for the control group were adolescents in the permanent dentition, 19 years of age, with no history of or ongoing orthodontic treatment. Subjects in the control group were required to have no orthodontic treatment need according to the Swedish Dental Board index [18].

Exclusion criteria were patients currently undergoing orthodontic treatment and those of younger age. There were no restrictions regarding the orthodontic treatment time nor the year of completing the orthodontic treatment. In this study, the subjects in the orthodontic group had finished their orthodontic treatment between the years of 2013 and 2022. Most of them had an orthodontic treatment duration of one and a half to 2 years. In the current study, the participants were predominantly male as a result of random selection of patients during retention control or annual dental checkups. Patient recruitment was consecutive, with participants attending the clinic for purposes unrelated to study participation.

All participants in this study were patients of the Department of Dental Medicine, Division of Orthodontics and Division of Paediatric Dentistry at Karolinska Institutet in Huddinge. Patients in the control group, as well as some in the orthodontic group, were screened during their regular dental checkup at the Division of Paediatric Dentistry. The remaining patients in the orthodontic group were screened at the Division of Orthodontics.

Thirty subjects filled out the questionnaire in the clinical setting following their regular dental checkup between May 2019 and September 2021. The subjects were required to have either already reached the age limit or to be turning 19 years old within the study year. Accordingly, subjects aged 18 years who would reach 19 years of age later that year were eligible for inclusion. Of these 31 and 32 initial patients, one from the orthodontic group and two from the control group were excluded from the analysis as they did not fulfill the inclusion criteria (one subject- 15 years old, two subjects- 16 years old). Nevertheless, in order to create two equal groups, two subjects aged 17 and 22 years, respectively, remained in the control group of this study.

**Figure 1:**
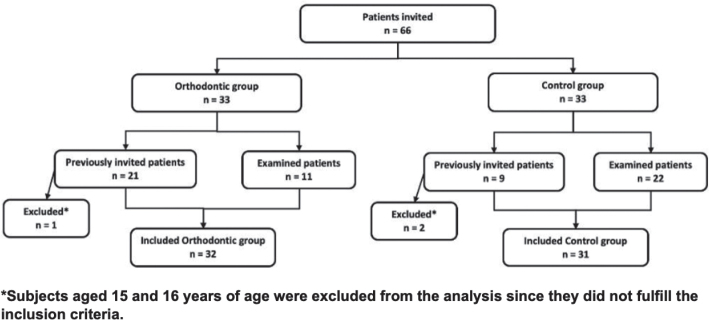
Flowchart describing study sample formation.

Thirty-three subjects were invited to participate during their annual dental checkup at the Division of Paediatric Dentistry of Karolinska Institutet between February 2022 and January 2023 (11 subjects in the orthodontic group and 22 subjects in the control group) (Figure 1).

### Clinical considerations

To collect data on the decayed, missing and filled teeth index (DMFT) and to verify earlier orthodontic treatment, information was collected from earlier patient records. Orthodontic treatment outcome was not clinically evaluated. Teeth that were missing due to orthodontic indications were not considered as ‘missing’ in the calculation of the DMFT.

### Questionnaire

The questionnaire consists of three parts: OHIP-14, JFLS-20 and OES-8.

First and foremost, the subjects were verbally informed about how they should interpret the ordinal scales of the questionnaire. It was important to explain the difference between 0- ‘never’ and ‘not applicable’, as the latter indicates that the respondent chose not to answer the question. Participants were encouraged to ask the examiner for clarification if they did not fully understand a question. The subjects start the questionnaire by rating their general health as well as their oral health, using a five-point scale (1- ‘excellent’, 2- ‘very good’, 3- ‘good’, 4- ‘quite poor’, 5- ‘poor’). In this questionnaire, the only personal information collected from the subjects was their age and gender (1- ‘girl’, 2- ‘boy’, 3- ‘none of the above’).

In the OHIP-14 index, subjects rate how frequently they experience the impact of each question using a five-point scale (0- ‘never’, 1- ‘hardly ever’, 2- ‘occasionally’, 3- ‘fairly often’, 4- ‘very often’) or may select ‘not applicable’. The OHIP-14 measures four dimensions of oral health, including oral function (Q1, Q2, Q4, Q7, Q8), orofacial appearance (Q5), orofacial pain (Q3) and psychosocial impact (Q6, Q9, Q10, Q11, Q12, Q13, Q14) (6, 19). The total score for OHIP-14 can range from 0 to 56, with 56 representing the highest negative score on OHRQoL.

In the jaw function index, subjects rate their limitation, in the last month, using a numeric rating scale (0- ‘no limitation’, 10- ‘extreme limitation’) for each item. The 20 items assess mastication (item 1–6), jaw mobility (items 7–10) along with verbal and emotional expressions (items 11–20). Miscellaneous functions, such as swallowing and yawning, are included in verbal and emotional expressions [8]. The total score can vary from 0 to 200, with 200 -the highest score – indicating severe functional limitations of the jaw.

In the aesthetic scale, subjects rate each question using a numeric rating scale (0- ‘very dissatisfied’, 10- ‘very satisfied’) or may select the alternative ‘not applicable’ if they choose not to answer. The questions include self-evaluation of the appearance of the face (Q1), facial profile (Q2), mouth (Q3), teeth alignment (Q4), tooth shape (Q5), tooth colour (Q6), gums (Q7), followed by a separate question regarding the general judgement of the orofacial components (Q8). The total score can vary from 0 to 70 for Q1–Q7, where 70 means aesthetic satisfaction and additionally a maximum of 10 points for Q8 [9].

### Statistical analysis

Power analysis was performed using IBM SPSS Statistics for Windows version 27 (IBM Corp., Armonk, NY, USA) to determine the required sample size for each group. Based on a previous study, to achieve a power of 80% with a significance level of α = 0.05, a sample size of 29 patients was needed in each group [19].

Data from the questionnaires were also analysed using the IBM SPSS Statistics version 27. The level of significance was set at *p* < 0.05 for all tests. For testing between the groups and between genders, the Mann–Whitney U test was used.

Spearman’s correlation test was used to analyse any correlation between total OHIP and DMFT. Linear regression analysis was used to determine the strength of association between the categorical variable (gender) and the scale- or interval-level variable (test score). Some descriptive data are presented as medians and ranges (confidence intervals) instead of means and standard deviations.

## Results

There were no dropouts in this study as no participant reported regretting their participation.

### Background data

The majority of the patient cohort were male (*n* = 39; 61.9%), which was also observed in the orthodontic group (*n* = 18; 56.3%) and the control group (*n* = 21; 67.7%). The median age was 19 years in both groups, with a range of 18–19 years in the orthodontic group and 17–22 years in the control group.

The mean age at completion of orthodontic treatment for the participants in the orthodontic group is presented in Table 1, with a range of 13 and 19 years old (Table 1).

**Table 1:** Descriptive data for the orthodontic group and the control group, respectively.

	Subjects	Female	Male	Non-binary	Mean age (y) (orthodontic treatment completed)	Minimum age (y) (orthodontic treatment completed)	Maximum age (y) (orthodontic treatment completed)
Orthodontic group	32	14	18	0	16.5	13	19
Control group	31	9	21	1	NA	NA	NA

NA: Not applicable since the subjects of the control group did not undergo any orthodontic treatment.

### Variables

**Figure 2:**
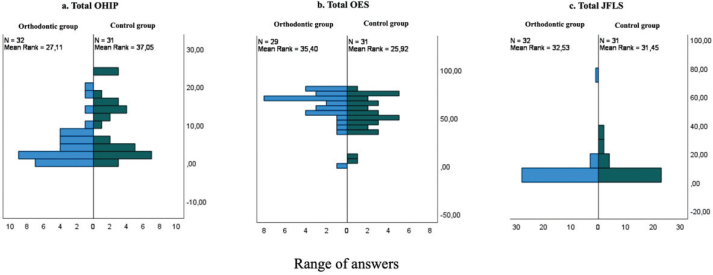
Spread of answers for all patients in the orthodontic group and control group for OHIP-14, OES [1–7] and JFLS-20, respectively. OHIP: Oral Health Impact Profile; JFLS: Jaw Functional Limitation Scale; OES: Orofacial Aesthetic Scale.

The total score of OHIP-S14 showed statistically significant differences between the orthodontic group and control group (*p* = 0.03) (Table 2 and Figure 2a). Subjects in both groups reported low scores, but subjects in the control group reported higher scores than those in the orthodontic group. When divided into the four domains of OHIP-14, only the psychosocial domain showed a statistically significant difference between the groups (*p* = 0.006).

**Table 2:** Mean (SD) of total oral health impact profile (OHIP-14); oral aesthetic scale (OES) and Jaw Functional Limitation Scale (JFLS) scores and difference between the orthodontic group and the control group.

	Orthodontic group (*N* = 32)	Control group (*N* = 31)	*P*-value
Total OHIP -S14	4.28 (5.12)	8.39 (7.66)	0.03*
Oral function	1.35 (1.6)	2.07 (2.3)	0.1
Orofacial pain	0.61 (0.76)	0.67 (0.84)	0.47
Psychosocial impact	2 (3.2)	5.25 (4.81)	0.06*
Orofacial appearance	0.32 (0.74)	0.73 (0.82)	0.19
Total OES (Q1-7)	56.57 (10.81)	48.76 (14.38)	0.028*
Global overall view of total aesthetic (Q8)	8.11 (1.59)	7.32 (2.38)	0.08
Total JFLS-20	4.84 (12.94)	5.96 (9.93)	0.8
Mastication	1.31 (2.37)	2.12 (3.62)	0.06
Jaw mobility	0.75 (1.81)	1 (2.85)	0.22
Verbal and emotional expressions	2.78 (10.26)	2.83 (5.65)	0.947

* Statistically significant *p* < 0.05.

*N*: total number of subjects sampled.

There were significant differences between the groups when comparing the total score of the OES (*p* = 0.036) (Table 2 and Figure 2b). In both groups, scores showed a wide range. However, in general, subjects in both groups more frequently chose lower scores regarding their tooth colour on the sixth question of the OES questionnaire. Four subjects in the orthodontic group and two subjects in the control group reported ‘not applicable’ for all or many of the questions of the OES questionnaire.

The JFLS-20 total score showed non-significant differences between the groups (*p* = 0.8) (Table 2 and Figure 2c). Six of the subjects in the orthodontic group and five subjects in the control group answered ‘not applicable’ on the JFLS-20 questionnaire. In general, the two groups reported low scores. A low score indicates no functional limitation, whereas a high score indicates greater functional limitation (possible range 0–200). No significant differences were found regarding the three subdomains of mastication, jaw mobility and verbal and emotional expression.

**Figure 3:**
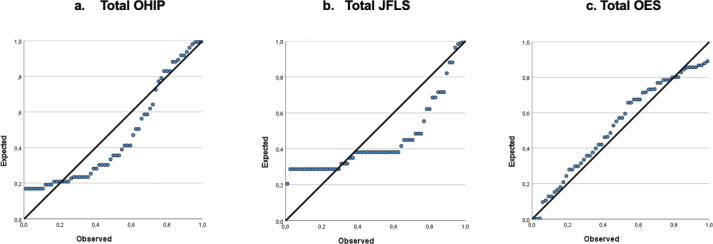
Linear regression analysis between gender and test score for (a) total OHIP, (b) total JFLS and (c) total OES. OHIP: Oral Health Impact Profile; JFLS: Jaw Functional Limitation Scale; OES: Orofacial Aesthetic Scale.

There were weak associations between gender and the different parts of the questionnaire (OHIP, JFLS-20 and OES) when tested with linear regression analysis (*R* ^2^ = 0.008, 0.03 and 0.01) (Figure 3 and Table 3). There was no correlation found between the DMFT and the OHIP, OES and JFLS-20, in both the orthodontic and control groups (Table 3).

**Table 3:** Linear regression analysis for both gender and DMFT between the different questionnaires.

Terms	SE	*R*	*R* ^2^	*P*-value	Lower 95% CI	Upper 95% CI
**Gender**						
Total OHIP	1.76	0.89	0.08	0.48	–2.29	4.75
Total JFLS	2.95	0.17	0.03	0.18	–1.91	9.92
Total OES	2.96	0.98	0.01	< 0.001*	–13.24	6.02
**DMFT**						
Total OHIP	0.29	0.15	0.02	0.2	–0.23	0.93
Total JFLS	0.5	0.17	0.03	0.16	–0.3	1.69
Total OES	0.06	0.14	0.22	16.20	–2.48	0.72

DFMT: decayed, missing and filled teeth index; OHIP: Oral Health Impact Profile; JFLS: Jaw Functional Limitation Scale; OES: Orofacial Aesthetic Scale.

* Statistically significant *p* < 0.05.

## Discussion

The main finding of this study is that earlier orthodontic treatment has a positive impact on the OHRQoL. This aligns with earlier studies reporting that patients who have undergone orthodontic treatment demonstrate improved OHRQoL [20, 21].

Nineteen-year-old patients, in the two groups, ranked their general health and oral health as high. Many subjects in the orthodontic group rated their oral health as ‘very good’, whereas many in the control group rated it as ‘good’. Overall, subjects in the orthodontic group more often reported higher ratings of their oral health.

There was a significant difference in the total OHIP-14 between the groups, indicating better OHRQoL for the orthodontic group. However, both groups reported relatively low scores throughout the OHIP-S14 questionnaire, indicating that only some questions were relevant to the patient. This finding aligns with a previous systematic review by Andiappan et al. [21], which concluded that orthodontic treatment resulted in lower OHIP-14 scores in adolescents older than 15 years.

Another finding is that the psychosocial impact domain is the one concerning the patients the most. This is in line with previous findings, where children and parents rate satisfying aesthetics as an essential component for psychosocial wellness [22]. A previous study has mentioned the importance of the psychosocial dimension being accounted for in the OHIP [23].

Regarding the OES total score, there are significant differences between the orthodontic group and the control group, with respect to their thoughts on their teeth, mouth and face but not with respect to the patient’s global assessment of orofacial aesthetics. A total of six subjects answered ‘not applicable’ to all or some questions of the OES questionnaire, which might indicate that patients are uncomfortable responding to questions related to their appearance. The OES indicates that the 19-year-old patients in the control group are more negatively affected by their aesthetic appearance than those in the orthodontic group. Some aspects of the OES do not have a direct correlation with orthodontic treatment, for example tooth colour. Tooth colour may influence an adolescent’s well-being, which in turn could affect the outcome of the OES questions. However, previous studies show that tooth colour has a weak effect on their social and emotional dimensions [24]. On the other hand, other studies report that tooth colour is the most common smile component causing dissatisfaction with teeth [25]. Therefore, further research is needed regarding this issue. Another aspect is the orofacial appearance, where a previous study state that patients with various conditions requiring aesthetically related treatment showed a moderate impact on the orofacial appearance dimension of OHRQoL [26]. However, as expected, the patients in our study did not express any concerns regarding their orofacial appearance. Regarding the aspect of dental aesthetics, a previous study stated that dental anomalies of the dentition affect the aesthetics of a smile [27]. It could be argued that the patients in our orthodontic group did not experience these problems after treatment since they are already treated for their malocclusion. The answers to the JFLS-20 showed no significant difference between the orthodontic group and the control group. Many participants in both groups reported low scores on the JFLS-20 questionnaire. This finding is in accordance with previous studies that found that, currently, there is insufficient evidence that individuals with orthodontic treatment history show better dental health and functionality [3, 19]. However, a difference was observed on an individual basis, with one subject in the orthodontic group and one subject in the control group expressing functional limitations.

In our study, no gender differences were observed in the questionnaire responses. The result is not in line with previous studies, which found that girls were less satisfied with their dental appearance than boys [28]. This could partially be explained by the uneven gender distribution between males and females in the orthodontic and the control group. The control group only contained nine females, while the orthodontic group had 14 females.

Regarding DMFT, we found no significant differences between the two groups when assessed in relation to total OHIP, total function and total aesthetic, respectively. This finding is supported by an earlier study in which DMFT was found to have no impact between the orthodontic and control group [19]. Patients in both groups had low DMFT scores, which may explain the lack of observable influence. It is possible that individuals with high DMFT are not eligible for orthodontic treatment because of their caries activity [29]. If the participants had a high DMFT value, it is possible that a difference would have been observed, as other studies has shown that caries have a negative impact on OHRQoL [30].

By the age of 19, it is also possible that the subjects may have forgotten the severity and effect of their former malocclusion, since OHRQoL changes with age. On the other hand, awareness of malocclusion and importance of oral aesthetics may emerge with age [10].

### Study strength and limitations

The strength of this study is that it uses three assessment tools to assess OHRQoL among 19-year-old patients. The combination of these three assessment tools allows us to identify correlations and understand how the subjects’ malocclusion affects their well-being.

A limitation of this study is that three different examiners performed the dental checkups on the subjects, while five examiners administrated the questionnaire and provided instructions. This may have resulted in different DMFT values, as dentists can differ in how they diagnose initial and manifest caries lesions [31]. Moreover, the involvement of multiple examiners may have influenced how the subjects interpreted the response options in the questionnaire, as there is a possibility that not all subjects received the same information.

Another limitation is the uneven distribution of males and females in the orthodontic and the control group. This may partially explain why no gender differences were observed in the questionnaire responses.

### Future applications

Since this study has shown a correlation between orthodontic treatment and both psychosocial and aesthetic impact, there is a need to continue research in this area. This study shows the importance of developing indices that take into account the psychosocial need for orthodontic treatment in relation to malocclusion.

Future studies could consider the orthodontic and control groups alongside a third group that includes 19-year-old patients with craniofacial anomalies, such as cleft lip and palate. A previous study found that OHRQoL in children was affected by oral clefts [32]. Including this third group would allow investigators to analyse the effect of treated cleft lip palate on the QoL in 19-year-old individuals.

In future studies, it would be valuable assessing whether the control group participants perceive a need for orthodontic treatment, although not enough to give a score in the treatment need indices, as this may influence their responses to the OES questionnaire. As Thiruvenkadam et al. [33] reported lower QoL among individuals seeking orthodontic treatment, this aspect deserves further investigation.

## Conclusion

The null hypothesis was rejected, as 19-year-old individuals who have had previous orthodontic treatment have significantly better OHRQoL when compared to 19-year-old individuals with no need for orthodontic treatment, specifically in the psychosocial and aesthetic domains.

## Supplementary Material



## Data Availability

The questionnaire that was used for this article can be accessed in the supplemental materials of the study conducted by Sörensen et al. [19].
