# Supplementary material for: Comparison of oral health-related quality of life in a group of 19-year-old patients with or without previous orthodontic treatment
Source: Acta Odontol Scand. 2026 Mar 4;85:45522. doi: 10.2340/aos.v85.45522 (PMC12966817; doi:10.2340/aos.v85.45522)
Supplement: Supplementary file 1 [file AOS-85-45522-s1.pdf]

Supplementary material has been published as submitted. It has not been copyedited or typeset by Acta Odontologica Scandinavica.

TREATMENT NEED INDEX translated to English
Issued by the Swedish National Board of Health and Welfare

OBJECTIVE INDEX				MALOCCLUSION
1	2	3	4	
			4	CLP (cleft lip and palate) and syndromes
				**Sagittal**
			4	Extreme postnormal (Class II) occlusion
1				Postnormal (Class II) occlusion without other anomalies
			4	Extreme prenormal (Class III) occlusion
		3		Prenormal (Class III) forced bite
1				Prenormal (Class III) occlusions with little negative overjet
				**Vertical**
		3		Deep bite with gingival irritation
	2			Deep bite with gingival contact but without gingival irritation
1				Deep bite without gingival contact
		3		Extremly open bite
1				Open bite with little frontal opening
				**Transverse**
		3		Crossbite causing transverse forced bite
1				Crossbite without transverse forced bite
1				Anterior crossbite of single teeth without forced bite
		3		Scissorbite interfering with articulation
				**Space deviations**
		3		Severe frontal crowding
	2			Severe crowding
1				Mild crowding
		3		Severe frontal spacing
	2			Severe spacing
1				Mild spacing
			4	Extensive aplasia
				**Individual teeth**
			4	Retained upper incisors
		3		Retained canines
	2			Aesthetically and/or functionally disturbing proclined incisors
	2			Aesthetically and/or functionally disturbing retroclined incisors
		3		Aesthetically and/or functionally disturbing rotations
	2			Moderate frontal rotations
1				Mild rotations of only little aesthetic and/or functional significance
	2			Infraocclusion of deciduous and permanent teeth

The objective treatment need is weighed together with the subjective treatment need, which reflects the patient's and/or the parents' perception of the need for treatment. Graded from 4 (very urgent need) to 1 (little need).
